# Beyond School Avoidance: Recognising, Identifying, and Addressing Autistic Burnout in Children

**DOI:** 10.1192/bjo.2024.433

**Published:** 2024-08-01

**Authors:** Georgie Siggers, Becky Day

**Affiliations:** Starjumpz, Crowborough, United Kingdom

## Abstract

**Aims:**

Autistic burnout, a profound psychological state characterised by increased stress, exhaustion, and a decline in functional abilities, has begun to be documented in adults but remains under-recognised in children. This abstract aims to shed light on autistic burnout in children, particularly in the context of school avoidance, and calls for a comprehensive approach to recognition, understanding, and intervention in this area.

**Methods:**

A retrospective audit was conducted on the case notes of 20 children, all diagnosed with autism, who had been unable to attend school for at least three months. The audit involved compiling a checklist of symptoms commonly associated with autistic burnout. This checklist included: chronic exhaustion, loss of skills previously acquired, diminished interest in activities, heightened sensory sensitivities, social withdrawal, mood dysregulation, and physical complaints. The primary objective was to investigate the presence of symptoms typically associated with autistic burnout in these children. To achieve this, information regarding these symptoms was extracted from their case notes.

**Results:**

Age: 8 to 17 years, 10 boys, 10 girls. 90% of the children had an EHCP (Education Health Care Plan). 100% of the children experienced chronic exhaustion, loss of skills & interests, increase in sensory needs, social withdrawal, mood dysregulation and physical symptoms.

**Conclusion:**

The alarming trend of school refusal among autistic children is a phenomenon that merits close scrutiny, not only for its impact on the child's education but also for the broader implications including the significant burden on families. The uniformity in the reported symptoms across the group strongly indicates a shared underlying issue. In the context of autism, these symptoms align with what is known about autistic burnout. These symptoms can significantly impact the quality of life and daily functioning, including the ability to attend school. Understanding of these symptoms as part of autistic burnout could lead to better support strategies, accommodations, and potentially improved outcomes for autistic children who are refusing school. It necessitates a shift from a potentially punitive approach to one that is compassionate and accommodative, ensuring that strategies are in place to support autistic children's return to school when they are ready and able to do so. These findings highlight an urgent need for research into autistic burnout in children, recognition of this concept by health and education and a need to re-evaluate current educational practices and support systems for autistic children in school.

